# Knockdown of SUMO-activating enzyme subunit 2 (SAE2) suppresses cancer malignancy and enhances chemotherapy sensitivity in small cell lung cancer

**DOI:** 10.1186/s13045-015-0164-y

**Published:** 2015-06-11

**Authors:** Xiaoke Liu, Yong Xu, Zongguo Pang, Fuchun Guo, Qing Qin, Tao Yin, Yaxiong Sang, Chengjun Feng, Xiaoyu Li, Li Jiang, Pei Shu, Yongsheng Wang

**Affiliations:** Department of Thoracic Oncology, Cancer Center, State Key Laboratory of Biotherapy/Collaborative Innovation Center of Biotherapy, West China Hospital, Sichuan University, Chengdu, Sichuan People’s Republic of China; Department of Thoracic Oncology, Cancer Center , West China Hospital, Sichuan University, Chengdu, Sichuan People’s Republic of China; Department of Pathology, West China Hospital, Sichuan University, Chengdu, Sichuan People’s Republic of China

**Keywords:** SUMO-activating enzyme subunit 2, Small cell lung cancer (SCLC), Chemotherapy sensitivity

## Abstract

**Background:**

SUMO-activating enzyme subunit 2 (SAE2) is the sole E1-activating enzyme required for numerous important protein SUMOylation, abnormal of which is associated with carcinogenesis. SAE2 inactivation was recently reported to be a therapeutic strategy in cancers with Myc overexpression. However, the roles of SAE2 in small cell lung cancer (SCLC) are largely unknown.

**Methods:**

Stably SAE2 knockdown in H446 cells were established with a lentiviral system. Cell viability, cell cycle, and apoptosis were analyzed using MTT assay and flow cytometric assay. Expression of SAE2 mRNA and protein were detected by qPCR, western blotting, and immunohistochemical staining. Cell invasion and migration assay were determined by transwell chamber assay. H446 cells with or without SAE2 knockdown, nude mice models were established to observe tumorigenesis.

**Results:**

SAE2 was highly expressed in SCLC and significantly correlated with tumorigenesis in vivo. Cancer cells with RNAi-mediated reduction of SAE2 expression exhibited growth retardation and apoptosis increasing. Furthermore, down-regulation of SAE2 expression inhibited migration and invasion, simultaneously increased the sensitivity of H446 to etoposide and cisplatin.

**Conclusions:**

SAE2 plays an important role in tumor growth, metastasis, and chemotherapy sensitivity of H446 and is a potential clinical biomarker and therapeutic target in SCLC with high c-Myc expression.

**Electronic supplementary material:**

The online version of this article (doi:10.1186/s13045-015-0164-y) contains supplementary material, which is available to authorized users.

## Background

Lung cancer is the first leading cause of cancer-related deaths in males while second in females all over the world [1, 2]. Small cell lung cancer (SCLC) accounts for 13 % of all newly diagnosed cases of lung cancer worldwide, representing approximately 180,000 cases per year [3–5]. Patients at extensive stage have median survival of 7–12 months, and 5-year survival is only 1–2 %. Whereas among patients at limited stage, median survival is about 23 months and 5-year survival is 12–25 % [6–10]. SCLC is the most aggressive type of lung cancer mainly due to rapid growth, wide invasion, and fast metastasis [8, 11–13]. Therefore, it is critical to investigate an effective strategy for SCLC treatment.

It is widely reported that SUMOylation is a post-translational modification, which is significantly involved in diverse cellular functions, including genome integrity, nuclear transport, gene expression, signal transduction, and cell proliferation and differentiation through modulating protein-protein interactions [14–19]. In addition, recent data have pointed that cancer is associated with alterations in SUMOylation [14]. Mechanically, SUMOylation requires three steps of enzymatic reactions to attach the small ubiquitin modifier (SUMO) protein to the substrates: activation with the E1 enzyme (SAE1/SAE2), conjugation with the E2 enzyme (UBC9), and ligation with E3 ligase. Especially, SAE2 is a critical component of the SUMO-activating enzyme which is necessary for SUMO pathway [15, 18–20]. Accumulating evidence indicates that SUMO pathway is involved in a variety of cancers [21–30]. A recent study showed that SAE2 inactivation could be a therapeutic strategy in Myc overexpression cancers [31]. However, the roles of SAE2 in SCLC, in which c-Myc was widely amplified and over-expressed [32–40], are still unknown.

Here, we investigated the role of SAE2 in SCLC. We found higher expression of SAE2 in SCLC than in normal tissues. Furthermore, we observed that down-regulation of SAE2 expression in SCLC cells suppressed cell proliferation, migration, invasion as well as tumor formation and promoted cell apoptosis. Based on these findings, we concluded that down-regulation of SAE2 expression enhanced tumor suppression and sensitivity of chemotherapy in SCLC, and targeting SAE2 may be a new method for patients with SCLC.

## Results

### Increased expression of SAE2 in SCLC patients and cell lines

**Fig. 1:**
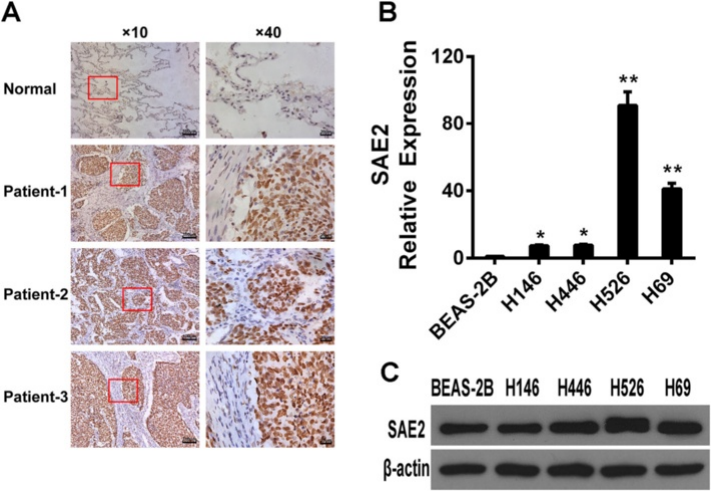
SAE2 expression in SCLC tissues and cell lines. **a** Representative immunohistochemical results of the expression of SAE2 in tumor tissues from SCLC patient (*n* = 20) and normal lung tissues (*n* = 5). **b** The expression of SAE2 mRNA in SCLC cell lines (H446, H146, H526 H69, and BEAS-2B). **c** The expression of SAE2 protein in SCLC cell lines (H446, H146, H526, H69, and BEAS-2B). Data represent means ± SEM of three independent experiments (*P < 0.05, **P < 0.01)

To investigate the roles of SAE2 in SCLC in which c-Myc was demonstrated to be widely amplified and expressed, we detected SAE2 protein level by immunohistochemical staining in the SCLC specimens and the normal lung tissues. Interestingly, we detected a significant elevated expression of SAE2 in SCLC tumor tissues(*P* < 0.001) (Fig. 1a). Moreover, we analyzed gene expression of SAE2 from the NCBI GEO database with 23 clinical small cell lung cancer (SCLC) samples from patients undergoing pulmonary resection and 42 normal tissue samples including the lung using Affymetrix Human Genome U133 Plus 2.0 Array (GSE43346). SAE2 was also highly expressed in SCLC compared to the normal tissues (Additional file 1: Figure S1). The mRNA and protein level of SAE2 were detected using quantitative real-time PCR and Western blot in several cell lines, including H446, H526, H69, H146, and BEAS-2B. Both mRNA expression and protein levels of SAE2 were significantly higher in SCLC cell lines compared with normal cell line (BEAS-2B) (Fig. 1b, c).These results indicated that SAE2 is highly expressed in SCLC tissues and cell lines.

### Inhibition of cell proliferation in H446 cells with SAE2 silence

**Fig. 2:**
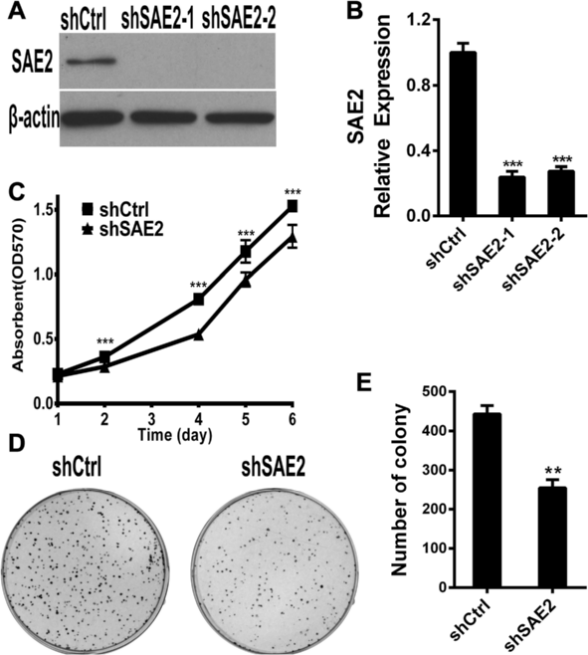
SAE2 affects the proliferation of SCLC cell line. Knockdown of SAE2 in H446 cell line confirmed by Western blot (**a**) and real-time PCR (**b**). **c** Growth rate of H446 cells with or without knockdown of SAE2 was determined by MTT assay. Data shown are means ± SD of three independent experiments. Representative colony images (**d**) and quantification of colony (**e**) are shown with or without knockdown of SAE2. Data are presented as means ± SD of three independent experiments (**P < 0.01, ***P < 0.001)

To investigate the role of SAE2 in SCLC, we firstly established H446 cells with stably down-expressing SAE2 (shSAE2-H446) by Plko.1-shSAE2. Cells stably harbored the corresponding empty Plko.1 vector which was established as control (shCtrl-H446). Quantitative real-time PCR and Western blotting analysis showed that the expression of SAE2 was markedly decreased in shSAE2-H446 cells (Fig. 2a, b). We further examined the effect of SAE2 on cell proliferation determined by the MTT assay. The growth rate revealed that silence of SAE2 significantly reduced viable cells (Fig. 2c). Consistently, less numbers of colonies were observed in shSAE2-H446 cells in colony formation assay (Fig. 2d), and the difference was significant (Fig. 2e).These results suggest that silence of SAE2 inhibits the growth of SCLC cell.

### Induction of apoptosis in H446 with SAE2 knockdown

**Fig. 3:**
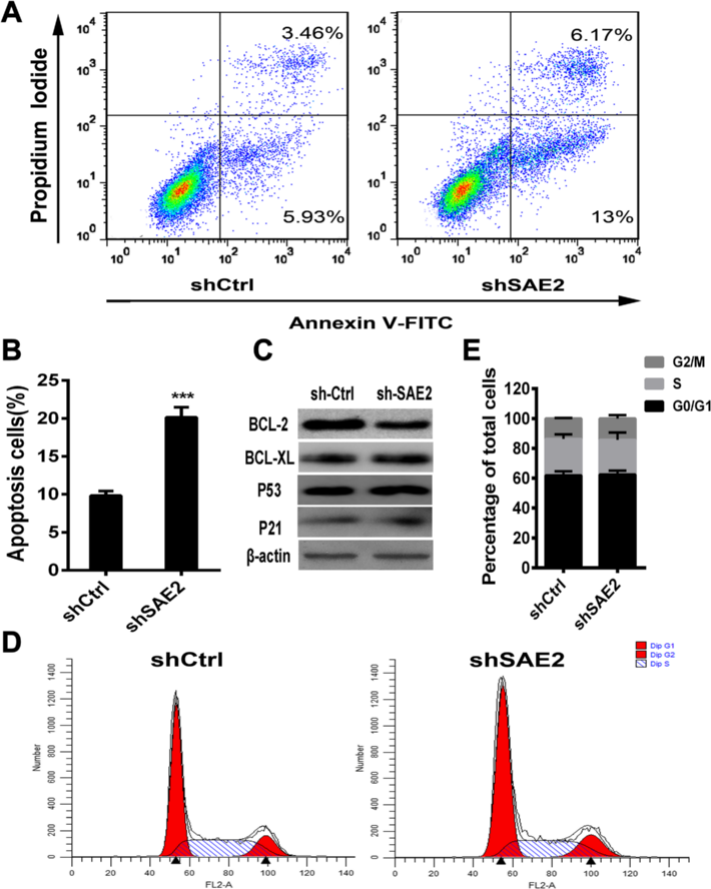
SAE2 is associated with apoptosis in SCLC cell line. **a** Representative FCM result stained by Annexin V-FITC and PI. Annexin V^+^/PI^−^ and Annexin V^+^/PI^+^ cells were designed as early stage and advanced stage of the apoptotic process. **b** The flow cytometry (FCM) results are presented as the percentage of apoptotic cells. The sum of FITC-positive cells in the *top right* and *bottom right* quadrants represents the total percentage of apoptotic cells. **c** Apoptosis-related protein levels were examined by Western blots using β-actin as a loading control. **d** Cell cycle analysis was performed by FCM and **e** the percentage of the cell population at different cell cycle phases was shown. Each data point represents means ± SD of three independent experiments (***P < 0.001)

To explore the effect of SAE2 deficiency on cell apoptosis and cell cycle, apoptosis assay by Annexin V-FITC/propidium iodide (PI) staining and propidium iodide (PI) staining were performed. Our results revealed that there were approximately 20 % apoptotic cells in shSAE2-H446 cells (Fig. 3a, second panel), compared to only 9.39 % of cells in shCtrl-H446 cells (Fig. 3a, first panel). Meanwhile, we detected proteins involved in apoptosis by Western blot. Expression of Bcl-2 was prominently decreased, while Bcl-XL, P53, and P21 were maintained (Fig. 3c). These data indicated that silence of SAE2 was sufficient to promote apoptosis by decreasing the expression of Bcl-2 in H446 cells. In addition, there was no significant difference in cell cycle of shSAE2-H446 cells compared with shCtrl-H446 cells after starving for 24 h, detected by PI staining (Fig. 3d, e). We conclude that knockdown of SAE2 in SCLC cells increased apoptosis.

### Knockdown of SAE2-inhibited cell invasion and migration in vitro and tumorigenesis in vivo

**Fig. 4:**
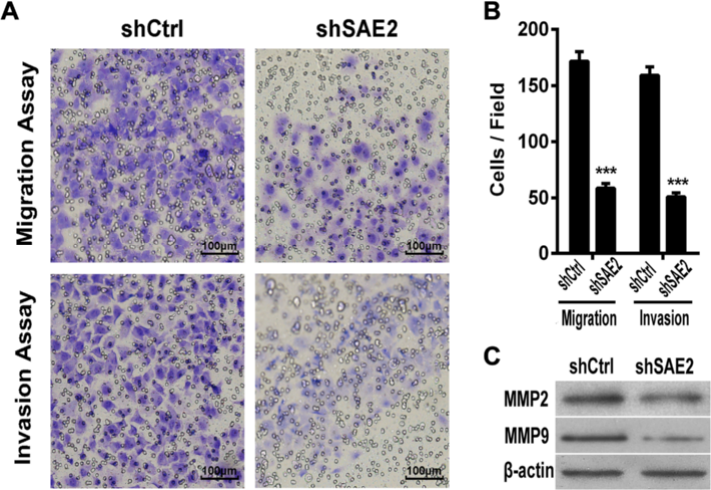
SAE2 is essential for the migratory and invasive potential of SCLC cells in vitro. **a** Transwell invasion and migration assays of shCtrl-H446 and shSEA2 H446 tumor cells were performed. **b** The results are showed as an average of the number of migration/invasion cells from six random microscopic fields. **c** MMP2 and MMP9 protein level was measured by Western blot. Each data point represents mean ± SD of three independent experiments (***P < 0.001)

**Fig. 5:**
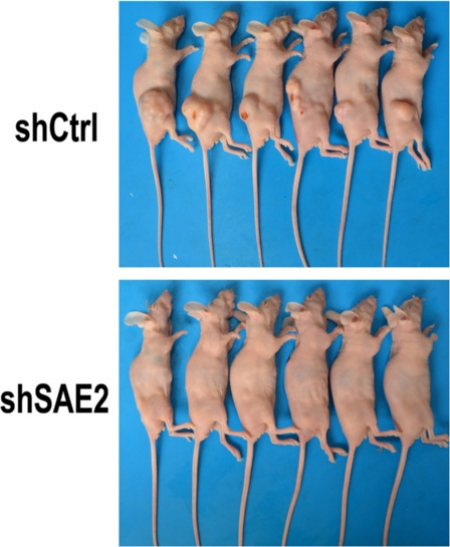
Effects of SAE2 on the tumorigenesis of SCLC cells. shCtrl-H446 or shSAE2-H446 cells were inoculated subcutaneously into the flanks of nude mice. The tumor in mice were observed for 8 weeks (*n* = 15)

We next investigated the effects of SAE2 on cell invasion and migration. A transwell cell migration assay showed that knockdown of SAE2 in H446 cells exhibited a significant decrease in cell migration ability (Fig. 4a). Furthermore, by using a transwell matrigel cell invasion assay, we found that the invasion ability of shSAE2-H446 cells was also significantly reduced (Fig. 4a, b). As MMP2 and MMP9 were crucial proteins involved in cancer cell metastasis, we reasoned that SAE2 might regulate MMP expression in the SCLC. Expression of MMP2 and MMP9 in shCtrl-H446 cells or shSAE2-H446 cells were measured by Western blot analysis. We found that the levels of MMP2 and MMP9 were decreased in shSAE2-H446 cells compared with that in shCtrl-H446 cells (Fig. 4c). These data indicated that silence of SAE2 was sufficient to inhibit invasion and migration by decreasing the expression of MMP2 and MMP9 in H446 cells. Furthermore, to test the effects of knockdown of SAE2 in vivo, shCtrl-H446 cells or shSAE2-H446 cells were inoculated subcutaneously into the flanks of nude mice, and the tumorigenesis in mice was observed for 8 weeks (Fig. 5). As the results shown in Table 1, the incidence of subcutaneous tumorigenesis in the athymic nude mice harboring shSAE2-H446 cells was 0/15 at 56 days post-inoculation, whereas 14/15 xenograft were established with shCtrl-H446 cells. These results demonstrated that knockdown of SAE2 markedly inhibited tumorigenesis of H446 cells in vivo.

**Table 1:** Effects of down-regulation SAE2 expression on subcutaneous tumor-forming rate in nude mice

Cell line	Incidence
shCtrl-H446	14/15
ShSAE2-H446	0/15

### Sensitization of chemotherapy in H446 cell line with SAE2 knockdown

**Fig. 6:**
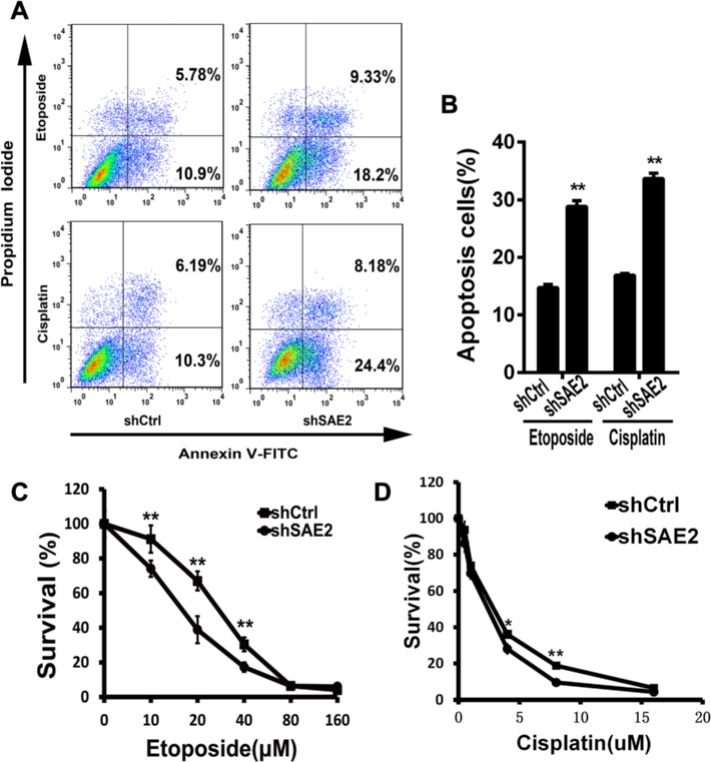
Knockdown of SAE2 sensitized chemotherapy in H446 (**a**). shCtrl-H446 and shSAE2-H446 tumor cells were treated with etoposide (15 μM) or cisplatin(1.5 μM) for 24 and 48 h, respectively. Cell apoptosis was detected by Annexin V and propidium iodide staining (**b**). The FCM results are presented as the percentage of apoptotic cells. Drug concentration–dependent cell survival curves for etoposide (**c**) for 48 h and cisplatin (**d**) for 72 h in shSAE2-H446cells or shCtrl-H446 cells. Each data point represents means ± SD of three independent experiments (*P < 0.05, **P < 0.01)

It is widely reported that SCLC is the most aggressive type of lung cancer mainly due to quickly refractory to initial therapy. We next investigated whether knockdown of SAE2 would sensitize SCLC cells to chemotherapy. Cell viability assay for stable cell lines shCtrl-H446 and shSAE2-H446 were performed after treatment with different concentrations of etoposide and cisplatin for 48 and 72 h, respectively (Fig. 6c, d). Our result showed that knockdown of SAE2 in H446 significantly reduced the IC50 of chemotherapeutical agents etoposide (16.65 μM in shSAE2-H446 vs 27.26 μM in shCtrl-H446) and cisplatin (1.874 μM in shSAE2-H446 vs 2.528 μM in shCtrl-H446) (Table 2). In our previous study, down-regulation of SAE2 inhibited cell growth mainly by inducing cell apoptosis, then we examined the apoptosis of SAE2 down-regulated cells treated with etoposide or cisplatin and showed that proportion of apoptotic cells was significantly increased in shSAE2-H446 cells (Fig. 6a, b). These results suggested that down-regulating SAE2 improved chemosensitivity in SCLC.

**Table 2:** IC50 values of chemotherapeutical agents

	IC50 value(μM)	
Cell line	Cisplatin	Etoposide
shCtrl-H446	2.528	27.26
shSAE2-H446	1.874	16.65

## Discussion

Although various efforts have been made to improve the treatment of SCLC, the patient prognosis has not been improved in the past decades [1, 7, 9, 10]. As an essential E1 activating enzyme for SUMOylation, SAE2 plays a key role in SUMOylation which is associated with several diseases including tumors [41]. Recent reports suggest that SAE2 deregulation induces the development of hepatocellular carcinoma [42]. Moreover, Kessler, JD et al. showed that SAE2 inactivation could be a therapeutic strategy in Myc overexpression cancers [31]. Therefore, we speculated that SAE2 was important for tumor formation and progression in SCLC which was characterized with high c-Myc expression. And we discovered an elevated expression of SAE2 in SCLC tissue and cell lines. We investigated the role of SAE2 in SCLC. Further, we provide the evidence that SAE2 plays an important role in regulating cellular proliferation, invasion, and sensitivity of chemotherapy in SCLC.

Several reports suggested that SUMOylation modification, occurs through a series of enzymatic reactions, is associated with apoptosis regulation, maintenance of genome integrity, modulation of subcellular transport, and transcription [17]. Therefore, the growth inhibition by knocking down SAE2 was assessed, and we discovered that selective down-expression of SAE2 significantly inhibit cell proliferation. Meanwhile, Annexin V-FITC/PI double staining showed an increased cell apoptosis when SAE2 was knocked down. Bcl-2, an important anti-apoptotic protein, was accordingly reduced in shSAE2-H446 stable cells, further implying that apoptotic pathway was involved in inhibition of cell proliferation. These data suggest that altered expression of SAE2 is an important contributor to the development of SCLC. Therefore, we analyzed the tumorigenesis of H446 with or without knockdown of SAE2. Xenograft model showed that selected down-expression of SAE2 significantly decreased tumorigenesis of H446. As demonstrated that misregulation of sumo proteins was involved in tumor development [42], our results suggest that the elevated expression of SAE2 in SCLC cells might contribute to tumorigenesis. This is likely due to the fact that SAE2 is a crucial enzyme for SUMOylation and numerous important proteins, such as tumor oncoproteins.

SCLC has the tendency for early dissemination and metastases [11]. And increasing data have indicated that SUMO modifications were critical regulators in cancer-related metastasis [23, 30, 41]. In our study, cell migration and invasion assay verified that down-expression of SAE2 inhibited the migratory and invasive properties of SCLC cells in vitro. MMP-2 and MMP-9 are correlated with invasion and metastasis through degradation of type IV collagen which is principal for the basement membrane [43, 44]. Here, we indicated that expression of MMP-2 and MMP-9 was decreased in shSAE2-H446 cells, which suggested that SAE2 silence-induced inhibition of SCLC invasion and migration might be related with MMP-2 and MMP-9. However, the molecular mechanism of shSAE2-H446-mediated inhibition of the invasion and metastasis in SCLC needed further research.

Treatment in SCLC is often associated with rapid drug resistance [5, 11]; therefore, new approaches to improve the efficiency of chemotherapy are extremely needed to be developed. Several studies have suggested that small ubiquitin like modifier SUMOylation is significantly involved in multidrug resistance in several cancers, such as ovarian carcinoma and hepatocellular carcinoma [26, 45, 46]. Consistently, we observed that down-expression of SAE2 significantly sensitized cells to cisplatin and etoposide in vitro. Annexin V-FITC binding assay proved that chemotherapy induced increased cell apoptosis in SAE2 knocked down cells. This may be due to SAE2’s role in signal transduction pathways including cytokines, Wnt, NF-κB, and growth factors [18, 42, 47].

Though SAE2 may be a candidate in SCLC treatment, further study needs to be done, especially considering its role dependent on Myc-driven cancers. JD Kessler reported that inactivation of SAE2 inhibited tumorigenicity of Myc-dependent tumors, SUM159 and MDA-MB-231, whereas MCF7 and SKBR3, both of which were Myc-independent, were unaffected. In addition, clinical data showed that expression level of SAE2 correlated with outcome in patient with Myc-high tumors but not Myc-low tumors. We also detected the expression of c-Myc in SCLC cells and found that H446, but not H69 and H526, displayed high expression levels of c-Myc (Additional file 1: Figure S2). Furthermore, silence of SAE2 with siRNA in H526 cells did not induce apoptosis (Additional file 1: Figure S3). This suggests that targeting SAE2 mainly plays a role in SCLC with high c-Myc expression.

## Conclusions

In summary, unprecedentedly, our studies confirmed that SAE2 was aberrantly overexpressed in SCLC significantly correlating to tumorigenesis. Meanwhile, knockdown of SAE2 not only negatively influenced the proliferation, migration, and invasion of SCLC cells but also facilitated basal apoptosis and chemotherapy-induced apoptosis. These findings demonstrate a crucial role of SAE2 in the progression of SCLC and suggest that SAE2 may serve as a clinical biomarker and therapeutic target in SCLC with high c-Myc levels.

## Methods

### Cell culture

Four human SCLC cell lines (H446, H146, H526, and H69) and one normal cell line (BEAS-2B) were used in this study. H446, H146, H526, H69, and BEAS-2B cell lines were cultured in RPMI-1640 Medium containing 10 % fetal bovine serum with 1 % penicillin/streptomycin (SIGMA) at 37 °C in a CO_2_ incubator (5 % CO_2_). Cells in exponential growth phase were used for all experiments.

### Immunohistochemical staining

All the tumor samples of 20 patients with SCLC and 5 normal lung tissues from West China Hospital were fixed in 10 % paraformaldehyde, embedded in paraffin, and cut in 5 μm serial sections. Immunohistochemical staining was performed using a peroxidase-labeled streptavidin-biotin technique. Firstly, tissue sections were deparaffinized and rehydrated. Then, sections were boiled in 10 mM sodium citrate buffer (pH 6.0) and maintained at a sub-boiling temperature for 10 min to retrieve antigenicity and were treated with 3 % H_2_O_2_ in methanol for 10 min to quench endogenous peroxidase activity. After washing in 10 mM PBS (pH 7.6), sections were incubated with 10 % normal mouse serum for SAE2 or rabbit serum (Solarbio Science and Technology) for c-Myc for 10 min to block nonspecific antibody binding. Sections were then incubated with mouse anti-human SAE2 polyclonal antibody (1:100) or rabbit anti-human c-Myc polyclonal antibody (1:100) overnight at 4 °C. After washing in PBS, sections were treated with a 1:100 dilution of biotinylated donkey anti-mouse IgG for SAE2 or anti-rabbit IgG for 30 min followed by a streptavidin-peroxidase conjugate for 30 min. A solution of 0.02 % diaminobenzidine hydrochloride (DAB) containing 0.03 % H_2_O_2_ was used as chromogen to visualize peroxidase activity. The preparations were lightly counterstained with hematoxylin, mounted with Permount (Thermo Fisher Scientific), and examined by light microscopy.

### Lentiviral vectors mediated SAE2-specific shRNA stable transfection

Before they were incubated overnight, 293 T cells were plated at 5 × 10^6^ cells per 100-mm dish. The cells were co-transfected with 9 μg pLKO.1-vector/pLKO.1-shSAE2, 9 μg psPAX2, and 4.5 μg pMD2.G-VSV-G (at a 2:2:1 ratio). Virus was harvested at 48 and 72 h post-transfection, filtered with a 0.45-μm pore size cellulose acetate filter (Millipore), and infected the H446 cells with the presence of 8 μg/mL of polybrene. Cells were selected with 0.9 μg/mL puromycin 24 h later.

### Western blotting

In brief, cells were washed three times with PBS buffer and lysed in RIPA lysis buffer (Beyotime) on ice. Total protein (50 μg/sample) was extracted and separated by 10 % sodium dodecyl sulfate-polyacrylamide gel electrophoresis (SDS-PAGE) and then transferred onto polyvinylidene difluoride (PVDF) membranes (Millipore, USA). Membranes were blocked with 5 % non-fat milk in TBST (10 mM Tris, pH 7.4150 mM NaCl, and 0.1 % Tween-20) at room temperature for 1 h. The blotted membranes were incubated with 1–2 μg/ml of primary antibodies (Anti-SAE2:ab118404, Anti-Bcl-2:CST#2870S,Anti-Bcl-XL:ab32370, Anti-P53:ab32049,Anti-P21:ab109199, Anti MMP2:ab92536, Anti-MMP9:ab3159) diluted in blocking solution at 4 °C overnight with gentle rocking. After washing five times with TBST, the membrane was reacted with the appropriate HRP-conjugated secondary antibody for 1 h at 37 °C. β-actin protein was also determined by using specific antibody (dilution 1:1000, Cell Signaling Technology) as a loading control. After extensive washing with TBST, proteins were visualized by the enhanced chemiluminescence (ECL) detection.

### RNA extraction and analysis by quantitative real-time PCR

Total RNA from each cell was extracted with RNA simple Total RNA Kit (TIANGEN BIOTECH, BEIJING). The RNA samples were reverse-transcribed into cDNA with the PrimeScript™ RT reagent Kit (TAKARA). Quantitative real-time PCR was conducted with Bio-rad CFX manager and SsoAdvanced SYBR Green Supermix (Bio-rad) as the detection dye. The primer sequences of PCR were as follows: SAE2, sense 5′-GATAACAGAGCTG CCCGAAAC-3′ and anti-sense 5′-ATAACACTCGGTCACACCCTTT-3′, GAPDH, sense5′GAAGGTGAAGGTCGGAGT-3′ and antisense 5′-GAAGATGGTGATGGGATTTC-3′. RT-PCR amplification was performed in 40 cycles with DNA denaturation at 95 °C for 5 s and annealing/extension at 60 °C for 20 s. For analysis, GAPDH mRNA was used to normalize RNA inputs, ΔΔCt values were calculated and converted to approximate fold change values (2-ΔΔCt).

### Cell proliferation assay

Seeded in 96-well plates were 2 × 10^3^ cells/well. From the second day to the sixth day, 20 μL MTT (Sigma-Aldrich) (5 mg/mL) was added to each well, incubated at 37 °C for 4 h, terminated with 150 μL of dimethyl sulfoxide (Sigma-Aldrich) per well, gently shook for 5 min, and determined with an ELISA reader (Bio-Rad) at 570 nm. For cell viable assay, cells were seeded in 96-well plates and exposed to various concentrations of etoposide (0, 5, 10, 20, 40, 80, and 160 μM) for 48 h or Cisplatin (0, 0.5, 1, 2, 4, 8, 16 μM) for 72 h; MTT assay was used to detect the chemotherapeutic sensitivity of cells. The concentration for 50 % of maximal effect (IC50 values) was calculated by GraphPad Prism.

### Annexin V-binding assay

Cells were seeded onto 6-well plates at a cell density of 2 × 10^5^ cells/well; 24 h later, cells were treated with Etoposide(15 μM) for 24 h or Cisplatin(1.5 μM) for 48 h. Control cells were treated with NS. Then cells were harvested and apoptosis was analyzed by flow cytometry using an FITC Annexin V Apoptosis Detection Kit I (BD Pharmingen) according to the manufacturer’s instructions.

### Cell cycle analysis

Cells were seeded at 3 × 10^5^ per well in 6-well plates and cultured with RPMI-1640 Medium non-containing fetal bovine serum for 24 h. Then cells were harvested and washed three times with cold PBS. Cells were fixed in 70 % ice-cold ethanol overnight, washed twice with PBS, stained with PI/RNase staining buffer (BD Pharmingen) at room temperature for 15 min, and detected by flow cytometer. Data were analyzed using CellQuest software.

### Cell migration assay

This cell migration assay was analyzed using transwell cell culture chambers (8 μm pore size) (Millipore). Briefly, cells (1 × 10^5^ /well) were serum starved for 24 h and placed in the upper chamber of a 24-well transwell in serum-free medium. RPMI 1640 containing 10 % FBS as chemoattractant were added to the lower chamber, and the cells were incubated at 37 °C in a CO_2_ incubator (5 % CO_2_) for 24 h. Then, the filter side of the upper chamber was cleaned with a cotton swab, fixed with 4 % formaldehyde for 10 min, and stained with 0.1 % crystal violet for 20 min. Finally, migrated cells were photographed under a light microscope and counted in six random microscopic fields.

### Cell invasion assay

Polycarbonate membranes of transwell chambers were coated with Matrigel (BD Biosciences) on the upper surface. Cells (3 × 10^4^/well) starved from serum for 24 h were resuspended in serum-free RPMI1640 and added to the upper chamber of 24-well transwell. And a complete RPMI1640 medium was added to the lower chamber as chemoattractant. The cells were incubated for 24 h at 37 °C with 5 % CO_2_. Non-invading cells were removed with a cotton-tipped swab from the top of the Matrigel. The invasive cells were photographed and counted in six random microscopic fields.

### RNA interference

siRNA for SAE2 was designed and synthesized by Life Technologies (Lifetech, China). The sequence of siRNA are as follows: siSAE2-1, sense:5′-GCAGCUGAUGUUC-CUCUUAdTdT-3′ and anti-sense:3′-dTdTCGUCGACUACAAGGAGAAU-5′; siSAE2-2, sense:5′-GCUGAGCUCAUAUGGGAUAdTdT-3′ and anti-sense:3′-dTdTCGACUCGAGUAUACCCUAU-5′. The sequence of negative control (siCtrl) was also designed by Life Technologies. H526 cells were plated onto a 6-well plate at 2 × 10^6^/well and transfected using GeneSilencer transfection Reagent (Genlantis, CA, USA) according to the manufacturer’s protocol. Cells were collected after 48 h for further experiments.

### Tumor formation rate following in vivo transplantation

A total of 30 Balb/c nude mice (6 weeks old) were obtained from the Vital River Laboratory Animal Technology, Beijing, housed in a specific pathogen free (SPF) environment; 7 days later, the mice were divided into two groups. The mice were injected with 1 × 10^7^ cells stably knocked down of SAE2 (shSAE2-H446) and parental pLKO.1-vector (shCtrl-H446) cells subcutaneously separately from each group. After inoculation, mice were housed in a sterile barrier system at constant temperature (25 ± 2 °C) and humidity (45-50 %). Tumor formation and growth were observed daily.

### Colony formation assay

Cells (single cell suspension) were seeded for colony formation assay in six-well plates at a density of 600 cells/well. The medium was replaced with fresh medium every 3 days. After 10 days, cell colonies were fixed and stained with crystal violet (0.5 % in 20 % methanol). Cell colonies were photographed under a light microscope and counted.

### Statistical analysis

Statistical analysis of SAE2 expression level of all 20 patients and 5 controls was performed using SPSS version 19.0 for windows (SPSS Inc) using Mann–Whitney test. All the other statistical analyses were performed using the GraphPad Prism 6.01 software program. Data analysis was carried out using the one-way ANOVA Tukey test for multiple groups and *t* test for two groups analysis. All data were summarized and presented as means ± SD or means ± SEM, *P* < 0.05 were considered to indicate statistically significant differences.

## Additional file

Additional file 1:
**Supplemental materials. Figure S1.** Expression levels of SAE2 in SCLC. **Figure S2.** Expression of c-myc in SCLC cells. **Figure S3.** Influence of SAE2 on apoptosis in H526 cells.

## Abbreviations

shRNA: short hairpin RNA
UTR: untranslated region
qRT-PCR: quantitative real-time PCR
MTT: 3-(4,5-dimethylthiazol-2-yl)-2,5-diphenyltetrazolium
SCLC: small cell lung carcinoma
FACS: fluorescence-activated cell sorting
PI: propidium iodide
PBS: phosphate-buffered saline
