# Supplementary material for: Knockdown of SUMO-activating enzyme subunit 2 (SAE2) suppresses cancer malignancy and enhances chemotherapy sensitivity in small cell lung cancer
Source: J Hematol Oncol. 2015 Jun 11;8:67. doi: 10.1186/s13045-015-0164-y (PMC4483218; doi:10.1186/s13045-015-0164-y)
Supplement: Additional file 1: — Supplemental materials. Figure S1. Expression levels of SAE2 in SCLC. Figure S2. Expression of c-myc in SCLC cells. Figure S3. Influence of SAE2 on apoptosis in H526 cells. [file 13045_2015_164_MOESM1_ESM.docx]

**Supplemental Materials**

**Fig.S1**

**
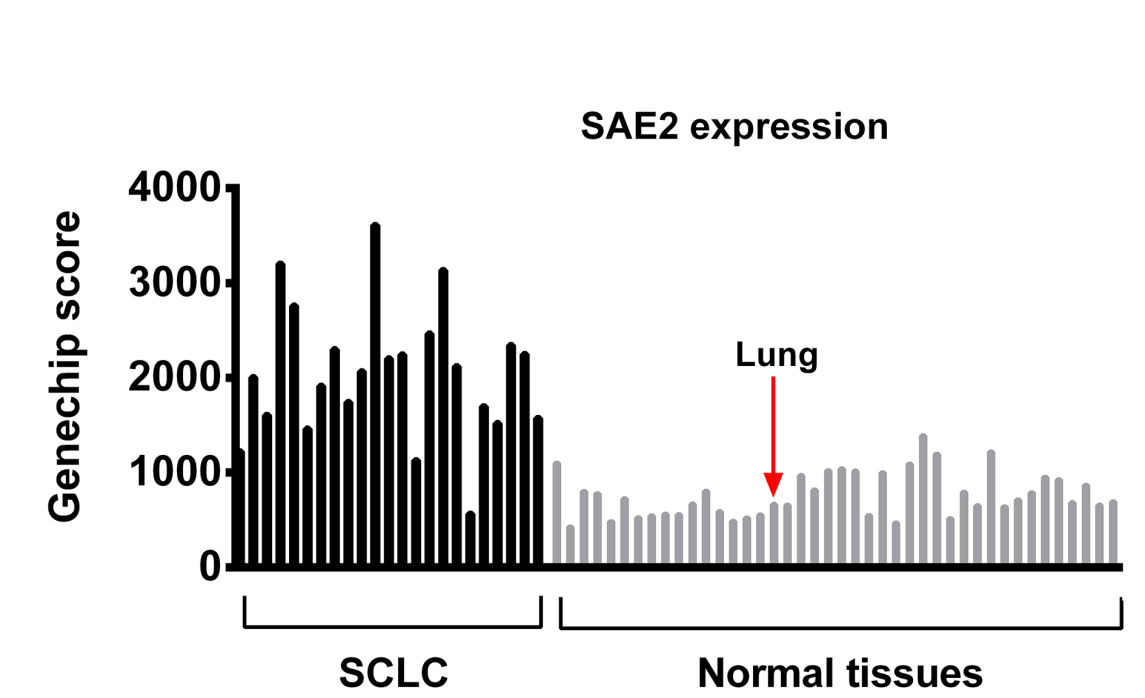
**

**Figure S1. Expression levels of SAE2 in SCLC.**

Gene expression of SAE2 from the NCBI GEO database ([GSE43346](http://www.ncbi.nlm.nih.gov/geo/query/acc.cgi?acc=GSE43346)) with 23 clinical small cell lung cancer (SCLC) samples and 42 normal tissue samples including the lung were analyzed and shown by Genechip Score.

**Fig.S2**


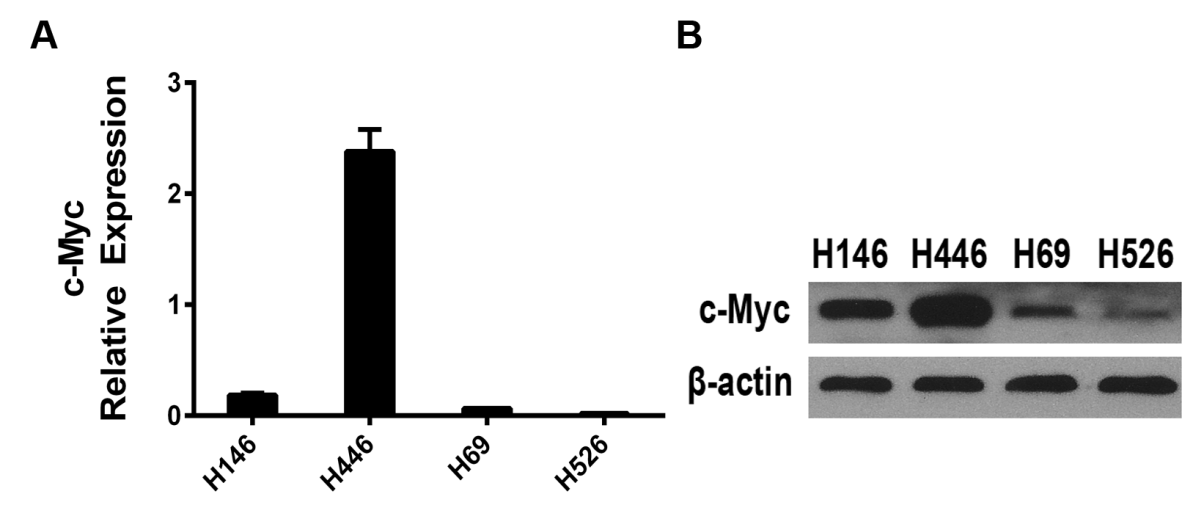


**Figure S2. Expression of c-myc in SCLC cells.**

Expression of c-myc were detected by western blot(B) and quantitative real-time PCR(A) in H146, H446, H69 and H526 cells.

**Fig.S3**

**
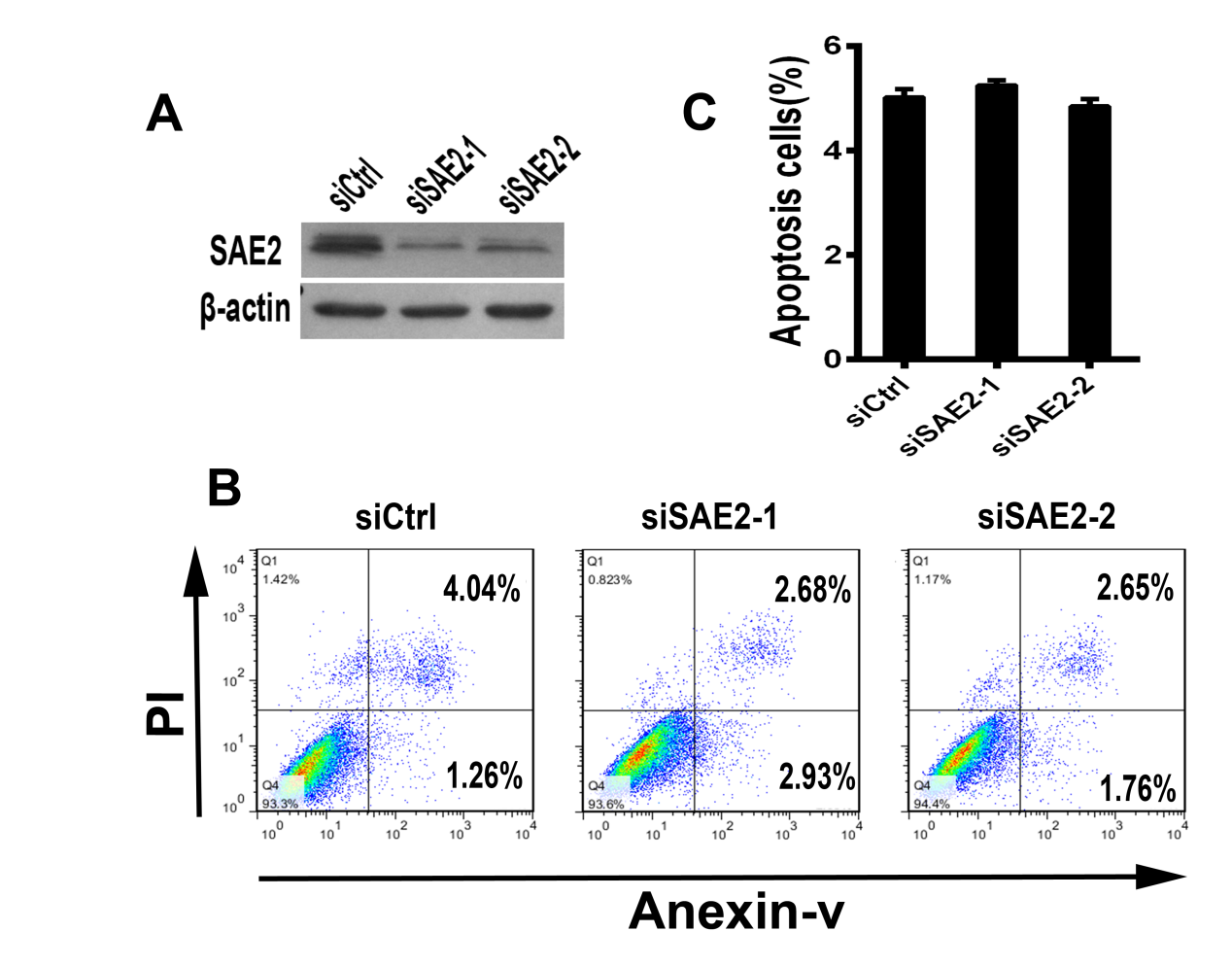
**

**Figure S3. Influence of SAE2 on apoptosis in H526 cells.**

(A) Protein expression of SAE2 were detected by western blot in H526 cells transfected with siRNA, β-actin served as the loading control. (B) Cells transfected with siSAE2 or siCtrl were collected and stained by Anexin V-FITC and PI. Apoptosis was determined by FACS analysis. (C) The results were presented as the percentage of apoptotic cells.
